# Carotid sinus hypersensitivity, as a cause of syncope, in a patient with coronary artery disease: A case report

**DOI:** 10.1016/j.amsu.2022.103940

**Published:** 2022-06-05

**Authors:** Bishal Dhakal, Nabin Simkhada, Bishnu Deep Pathak, Binaya Subedi, Dilip Thapa, Suchita Acharya, K.C. Prabhat, Parag Karki

**Affiliations:** a Nepalese Army Institute of Health Sciences, Sanobharyang, Kathmandu, Nepal; b Chitwan Medical College, Bharatpur, Chitwan, Nepal

**Keywords:** Carotid sinus hypersensitivity, Carotid sinus syndrome, Carotid sinus massage, Orthostatic hypotension, Vasovagal syncope

## Abstract

**Introduction:**

Carotid sinus hypersensitivity is one of the unexplained causes of syncope in old age. There are variations in presentations of carotid sinus hypersensitivity.

**Case presentation:**

A 56-year-old male, known case of coronary artery disease, presenting with syncope, was diagnosed as carotid sinus hypersensitivity finally. He was treated with the placement of dual chamber pacemaker.

**Clinical discussion:**

The etiology of unexplained syncope is itself a challenging for clinicians. In the presence of a known risk factor, it is uncommon for carotid sinus hypersensitivity to be present as a cause of syncope.

**Conclusions:**

Hence, the rare disorder like carotid sinus hypersensitivity should also be considered as a cause of syncope despite the presence of co-morbidity like coronary artery disease.

## Highlights

• Carotid Sinus Hypersensitivity is one of the causes of unexplained syncope in old age.
• Coronary artery disease is one of the etiological factors in unexplained syncope.
• Carotid Sinus Hypersensitivity is an uncommon cause of syncope in presence of coronary artery disease.
• The variations in carotid sinus hypersensitivity should be differentiated and treated accordingly.

## 1 Introduction

The etiology behind syncope is diverse. The carotid sinus hypersensitivity (CSH) is one of the causes for unexplained syncope in elderly patients. CSH along with the reproduction of syncopal event by carotid sinus massage has been referred to as carotid sinus syndrome (CSS). CSH is considered as a treatable cause of undiagnosed syncope [1]. Since Weiss and Baker described CSH in 1933, several studies have been conducted regarding its clinical relevance and management options [2]. The three varieties of CSH have been described in literature as cardioinhibitory, vasodepressor or mixed type [3].

The prolonged stimulation of carotid sinus has been seen to produce hypotension or syncope. The prevalence of CSH according to some studies has been shown in the range of 0–62% [4]. The prevalence of CSH is found to increase with age [5]. It accounts for about one-third of symptoms in older patients presenting with syncope [4].

Here, we report a case of a 56-year-old male presenting with loss of consciousness, having underlying co-morbidity as coronary artery disease (CAD). This created a diagnostic dilemma among the treating physicians regarding the cause of the syncopal event as eventually CSH was found to be the cause.

## 2 Case presentation

A 56-year-old Hindu male, known case of coronary artery disease (CAD), presented to our emergency department with a complaint of loss of consciousness (LOC). It was sudden in onset, lasting for a few minutes, and was preceded by sweating. There was a history of two episodes of LOC. It was not associated with any position or activity. There was no history of chest pain, palpitation, headache, weakness, abnormal body movements, fever, trauma, frothing at mouth, up rolling of eyes, tongue bite and tinnitus. He was non-smoker and non-alcoholic. There was no any relevant family history significant to the patient condition.

On clinical examination, he was hemodynamically stable, with normal blood pressure on supine (126/90 mmHg), and standing position (110/88 mmHg). There was no significant postural drop in blood pressure, ultimately ruling out postural hypotension. The general and systemic examinations were unremarkable. He was admitted in medical ward for further evaluation.

The baseline laboratory investigations are shown in Table 1.

**Table 1:** Baseline laboratory investigations.

Laboratory tests	Result	Unit	Reference range
Total Leukocytes Count	4.6	10^˄^3/μL	4–11
Neutrophil	70	%	40–80
Lymphocyte	20	%	20–40
Hemoglobin	13.7	g/dl	13–17
Platelet Count	137	10^˄^3/μL	150–450
Urea	19	mg/dl	17–43
Creatinine	1.0	mg/dl	0.7–1.3
Sodium	142	mEq/L	135–145
Potassium	4.0	mEq/L	3.5–5.5
Bilirubin Total	0.8	mg/dl	0.1–1.2
Bilirubin Direct	0.3	mg/dl	0.0–0.2
Alkaline Phosphatase (ALP)	48	U/L	53–128
Alanine Transferase (ALT)	34	U/L	0–35
Aspartate Transferase (AST)	32.7	U/L	0–35
Random Blood Glucose	99	mg/dl	70–140
Prothrombin time (PT)	17.3	seconds	11–13.5
CPK NAC	144	U/L	20–200
CPK MB	28	U/L	<35
Troponin I	Negative		
FT3	3.14	pg/ml	2.3–4.2
FT4	0.99	ng/dl	0.89–1.76
Thyroid Secreting Hormone (TSH)	5.10	μIU/ml	0.35–5.50
Total Cholesterol	201.4	mg/dl	0–200
High Density Lipoprotein (HDL)	32.3	mg/dl	40–60
Low Density Lipoprotein (LDL)	139.0	mg/dl	0–100
Tri-glyceride (TG)	129.3	mg/dl	0–180
Serum magnesium	2.98	mg/dl	1.8–2.6
Serum Phosphorus	2.81	mg/dl	2.4–4.4
Serum Calcium	9.21	mg/dl	8.6–11.8

On etiological workup for syncope, magnetic resonance imaging (MRI) of head showed a small locus of blood degradation product in the right frontal lobe. The electroencephalogram (EEG) showed normal background activity and was reactive to eye opening and closing. The carotid doppler showed normal scan. And, 2D echocardiography showed a reduced ejection fraction of 40% with no any outflow tract obstruction. These were the investigations done for etiological workup for unexplained syncope. At first, the etiology of syncope was considered to be coronary artery disease (CAD).

While the etiology for the syncope was in dilemma, an incidental carotid sinus massage resulted in sinus pause for more than 3 s in continuous electrocardiogram (ECG) monitoring. Carotid sinus massage (CSM) was performed sequentially over both left and right sides. It was performed both on supine and upright position for 5 s. The demonstration for sinus pause during CSM in ECG is shown in Video 1. And reversion to the normal rhythm after CSM is shown in Video 2. The differential diagnoses such as vasovagal syncope (VVS) and orthostatic hypotension (OH) were ruled out as the syncope was not associated with specific position or emotional and stressful events. He was then diagnosed as carotid sinus hypersensitivity (CSS). He was kept under medications as shown in Table 2.

**Table 2:** Medications.

Route	Drugs	Dose	Dosage
Tablet	Aspirin	75 mg	Once a day (OD)
Tablet	Clopidogrel	75 mg	Once a day (OD)
Tablet	Atorvastatin	20 mg	Hora Somnae (HS)
Tablet	Metoprolol	37.5 mg	Once a day (OD)
Tablet	Isosorbide Mononitrate	10 mg	Twice a day (BD)

Supplementary video related to this article can be found at https://doi.org/10.1016/j.amsu.2022.103940

The following is/are the supplementary data related to this article:

Multimedia component 1 Multimedia component 1

Multimedia component 2 Multimedia component 2

The definite treatment performed for CSH was dual chamber pacemaker placement. It was done in a heart center as the facility of cardiac catheterization was not available in our center. The pacemaker was placed through transvenous access via subclavian vein under local anesthesia. During the course on hospital, he was hemodynamically stable. He accepted the treatment and was recovering well. He was discharged on above mentioned medications after a week of stay in the hospital.

## 3 Discussion

The diagnostic approach for syncope is complex and multidimensional. CSH has been found as a cause of unexplained syncope in elderly patients for a long time now. CSH is defined as sinus pause for more than 3 seconds and/or fall in systolic blood pressure (SBP) of 50 mmHg or greater in response to CSM [6]. An asystole lasting for more than 3 seconds is termed as cardioinhibitory type of CSH. And, fall in systolic blood pressure (SBP) of 50 mmHg or greater is termed as vasodepressor type of CSH. If both of these are present, it is of mixed type. The term CSS is used when CSH is associated with spontaneous syncope. The diagnostic criteria for CSS is reproduction of spontaneous syncope during 10 seconds of CSM on both right and left carotid sinus sequentially [6].

The cardioinhibitory type is a common variant of CSH as compared to vasodepressor and mixed type. The vasodepressor type is a least common type of CSH [7]. It is important to distinguish between cardioinhibitory and vasodepressor type of CSH. During CSM, atropine is given in order to distinguish between the above mentioned two types. If the blood pressures still declines despite not having bradycardia, it is suggestive of vasodepressor type [1,2]. In our case, we could not delineate between cardioinhibitory and vasodepressor type due to technical infeasibility. We diagnosed it as CSH, cardioinhibitory type, as it was the commonest among the three.

The symptoms that patient can present with CSH include syncope or dizziness based on the perfusion to brain [7]. Carotid sinus massage is a simple and reliable test to diagnose CSH. But, possibility of errors has been described in performing CSM. The precise anatomical location and position of the patient are some of the factors for errors in performing CSH [2]. We performed CSM on both side in both positions sequentially. As there was no reproducible syncope during the massage, it did not fit into carotid sinus syndrome category.

According to a study, the symptoms are more profound in upright position as compared to supine position [8]. In elderly population, CSH has been a modifiable risk factor for non-accidental falls [9]. CSH has been shown to be associated with other causes of syncope like orthostatic hypotension (OH) and vasovagal syncope (VVS). A study by Maw Pin Tan et al., showed CSH, OH and VVS to be common causes for syncope in elderly and likely to co-exist in an affected individuals [10]. In our case, there was no coexistence of these entities.

Both the pharmacological and interventional management strategies are being practiced for CSH. Before the pacing therapy, treatment options included anticholinergic drugs like atropine, radiation therapy and carotid sinus denervation [3,11]. The preferred method of treatment that has been widely accepted is implantation of cardiac pacemaker [3,8,11,12]. As dual-chamber pacemaker activates both atria and ventricles, it is a treatment of choice in cardioinhibitory type of CSH [8]. The vasodepressor or mixed type can be treated with mineralocorticoids or radiation therapy and denervation of carotid sinus based on severity. Our case was treated with dual-chamber pacemaker placement.

There were certain limitations of our study. We could not distinguish between the two variants of CSH because of technical issues. It would be helpful in delineating the treatment pattern based on the variant. We could give atropine and monitor the vital parameters as described above for the differentiation. Similarly, we could not do follow up with the patient after discharge from the hospital. Despite having limitations, it provides insight for understanding uncommon etiology of unexplained syncope in the presence of significant risk factor.

## 4 Conclusions

CSH is one of the unexplained causes of syncope in old age. It may create a diagnostic challenge, especially in patients with underlying co-morbidity like CAD. As CAD is also a cause of syncope, it is uncommon to find CSH as a cause. So, the clinicians should be aware of this rare cause while attending a case of syncope even in the presence of comorbidity like CAD, and management should be planned accordingly.

## Ethical approval

This is a case report, therefore, it did not require ethical approval from ethics committee.

## Funding

The study did not receive any grant from funding agencies in the public, commercial or not-for-profit sectors.

## Author contribution

We the undersigned declare that this manuscript is original, has not been published before and is not currently being considered for publication elsewhere.

We confirm that the manuscript has been read and approved by all named authors and that there are no other persons who satisfied the criteria for authorship but are not listed. We further confirm that the order of authors listed in the manuscript has been approved by all of us.

We understand that the Corresponding Author is the sole contact for the Editorial process. He/she is responsible for communicating with the other authors about progress, submissions of revisions and final approval of proofs.

**Correspondence:** Bishal Dhakal, Nepalese Army Institute of Health Sciences, 44600 Kathmandu, Nepal. Email: swarnimdhakal@gmail.com, Phone: +977 9846491651.

Authors as follows:

1. Bishal Dhakal
2. Nabin Simkhada
3. Bishnu Deep Pathak
4. Binaya Subedi
5. Dilip Thapa
6. Suchita Acharya
7. Prabhat K·C
8. Parag Karki

Author 1: Contributed in data collection, literature review, writing the manuscript.

Author 2: The resident physician, who helped in the diagnosis and supervised in case presentation.

Author 3: Contributed in literature review and revising the manuscript.

Author 4: Contributed in manuscript review and data collection.

Author 5: Contributed in manuscript and literature review.

Author 6: Contributed to literature review and data collection.

Author 7: Contributed in data collection and literature review.

Author 8: Contributed in supervision, reviewing and editing manuscript.

## Registration of research studies

Not applicable.

## Guarantor

Bishal Dhakal, Nepalese Army Institute of Health Sciences, 44600 Kathmandu, Nepal. Email: swarnimdhakal@gmail.com, Phone: +977 9846491651.

## Consent

Written informed consent was obtained from the patient for publication of this case report and accompanying images. A copy of the written consent is available for review by the editor-in-chief of this journal on request.

## Provenance and peer review

Not commissioned, externally peer reviewed.

## Declaration of competing interest

The authors report no conflicts of interest.
